# Depletion of macrophages with clodronate liposomes partially attenuates renal fibrosis on AKI–CKD transition

**DOI:** 10.1080/0886022X.2022.2149412

**Published:** 2023-01-13

**Authors:** Zhizhi Hu, Juan Zhan, Guangchang Pei, Rui Zeng

**Affiliations:** Division of Nephrology, Department of Internal Medicine, Tongji Hospital, Tongji Medical College, Huazhong University of Science and Technology, Hubei, People’s Republic of China

**Keywords:** Ischemia/reperfusion injury, macrophage, renal fibrosis, clodronate liposomes

## Abstract

Clodronate liposomes are bisphosphonates encapsulated by liposomes that are known to induce macrophage depletion *in vivo*. In a previous study, clodronate liposomes improved renal ischemia/reperfusion (I/R) injury in mice, which may be due to effects on macrophage phenotypes. However, how inflammatory cytokines secretion participates is unknown. In this study, we investigated the effect of macrophages in the I/R kidney by depleting macrophages with clodronate liposomes and changing inflammatory cytokines. C57BL/6 mice underwent I/R injury with or without clodronate liposomes administration on Days 5 and 15. Tubular injury, collagen deposition, and fibrosis were detected and analyzed by histological staining, immunocytochemistry (IHC), flow cytometry (FACS), and reverse transcription–polymerase chain reaction (RT–PCR). Inflammatory cytokines were detected and analyzed by Western blotting and RT–PCR. We found that clodronate liposomes alleviated renal fibrosis and tissue damage on both Days 5 and 15. KIM-1, IL-10, and TGF-β were reduced significantly in the clodronate liposomes treatment group. However, TNF-α was not different between the clodronate liposomes treatment group and the phosphate-buffered saline treatment group on either Day 5 or Day 15. Thus, clodronate liposomes can alleviate renal fibrosis and tissue damage and reduce the inflammatory cytokines IL-10 and TGF-β, suggesting that clodronate liposomes alleviate renal fibrosis may because of M1/M2 polarization.

## Introduction

Acute kidney injury (AKI) is common in hospital patients and very common in critically ill patients, but no specific therapies have emerged that can attenuate AKI or expedite recovery [1,2]. Inflammation is well known to play a critical role in the pathophysiology of kidney ischemia/reperfusion (I/R) injury [3].

Macrophages play a very important role in multiple functions [4]. These cells can massively increase under inflammatory conditions, which is relevant for multiple diseases. Macrophages belong to the mononuclear phagocytic system, and plasticity and functional polarization are features of the mononuclear phagocyte system [5]. Traditionally, macrophages are subdivided into two subpopulations: classically activated or M1 macrophages and alternatively activated or M2 macrophages [6].

M2 macrophages secrete high amounts of interleukin 10 (IL-10) and transforming growth factor-β (TGF-β) to suppress inflammation, contribute to tissue repair, remodeling, and vasculogenesis and maintain homeostasis [7]. It has been demonstrated that macrophages shift their phenotype from pro-inflammatory M1 to anti-inflammatory, pro-resolving M2 macrophages, helping kidney repair following I/R injury [8].

Clodronate liposomes are bisphosphonates encapsulated by liposomes that are known to induce macrophage depletion *in vivo* [9]. With the encapsulation of the bisphosphonate clodronate into liposomes, an efficient reagent for the nonselective depletion of macrophages has been developed and successfully applied in several immunological studies [10]. Previous studies have also shown that nonselective macrophage depletion by clodronate liposomes administration reduces experimental acute kidney damage by diminishing persistent inflammation and the subsequent development of fibrosis [11]. Ferenbach et al. found that clodronate liposomes treatment induced marked functional protection and resulted in significantly reduced acute tubular necrosis [12]. These data indicate that the mechanism is independent of inflammatory cytokines secretion. However, how inflammatory cytokines secretion participates remains unknown.

In this study, we investigated the changes in renal injury or renal fibrosis in I/R injury by depleting macrophages with clodronate liposomes. Moreover, we also examined changes in inflammatory cytokines during clodronate liposomes treatment.

## Materials and methods

### Mice

Six- to 8-week-old male C57BL/6 mice (weight, 20–25 g) were purchased from Hua Fukang Company (Beijing, China). All animals were bred at Tongji Medical School, and all procedures were approved and performed in accordance with the institutional guidelines for animal care.

### Animal model

Fifty male C57BL/6 mice were randomly divided into five groups: sham operation group (Sham group, *n* = 10), injection of phosphate-buffered saline (PBS) 5 days after renal I/R group (Day 5 + PBS, *n* = 10), injection of clodronate liposomes drug 5 days after I/R group (Day 5 + CL, *n* = 10), injection of PBS 15 days after renal I/R group (Day 15 + PBS, *n* = 10), and injection of clodronate liposomes drug 15 days after I/R group (Day 15 + CL, *n* = 10). The PBS treatment group was divided into a Day 5 + PBS group and a Day 15 + PBS group. The clodronate liposomes treatment group was divided into a Day 5 + CL group and a Day 15 + CL group. Mice in the operation group were anesthetized by intraperitoneal injection of 1% sodium pentobarbital (0.2 mL/100 g), the back skin was prepared, disinfected, and a midline incision was made on the back, the left kidney was exposed, the renal capsule was separated, and the left renal pedicle was clamped for 30 min. The arterial clamp was released after 30 min, and the skin and tissue were sutured. Clodronate liposomes drug (200 µL) or PBS was injected *via* the tail vein soon after I/R surgery, and then 100 µL was injected *via* the tail vein every other day thereafter [12]. The specimen was taken 5 days and 15 days after clodronate liposomes drug or PBS injection. Sham animal models underwent a similar surgical procedure without clamping of the left kidney pedicle.

### Histopathology

The kidneys in different groups were weighed and lavaged with normal saline until the kidneys turned white. They were fixed in 4% paraformaldehyde for 24 h, embedded in conventional paraffin, sectioned at 3 μm, and stained with Periodic Acid Schiff (PAS), Masson’s Trichrome (Masson), and Sirius Red. Glomerulosclerosis and interstitial damage were observed under a light microscope. PAS was used to evaluate tubular injury, and Masson trichrome (Masson) and Sirius red staining were used to estimate renal interstitial fibrosis levels. The renal tubular damage score was based on tubular necrosis grade, cast formation, tubular dilation, and brush border loss, with scores corresponding to the following percentages of renal tubular damage: 0, 0%; 1, 10%; 2, 11–25%; 3, 26–45%; 4, 46–75%; and 5, ≥76%.

### Flow cytometry

In different groups, the kidney tissue of the mice was harvested, and the tissue was cut with scissors and digested with collagenase for 1 h. The cells were filtered through a 200-mesh filter, lysed with 3 mL of red blood cell lysis buffer for 5 min, and washed with PBS [13]. Cells were labeled for 30 min at 4°С in fluorescence activated cell sorter (FACS) buffer (PBS containing 2% FBS and 0.02% azide) with manufacturer-recommended concentrations of purified anti-mouse CD45-FITC (1:100, Biolegend, San Diego, CA, USA), F4/80-PE (1:100, Biolegend, San Diego, CA, USA), CD11b-APC (1:100, eBioscience, San Diego, CA, USA), and Gr-1-PE (1:100, eBioscience, San Diego, CA, USA) for 30 min. Cells incubated with FACS buffer alone were used as negative controls. The cells were analyzed by flow cytometry (FACSCalibur, BD Biosciences).

### Quantitative reverse transcription–polymerase chain reaction

One microgram of renal tissue total RNA was extracted using TRIzol according to the manufacturer’s instructions (Invitrogen, USA) and was reverse transcribed into cDNA according to the manufacturer’s instructions (Promega, USA). Polymerase chain reactions (PCR) were performed using a Roche light 480II using SYBR Master Mix (Qiagen, Germany). The ΔΔCt method was used for analysis. The PCR primer sequences are presented in Supplementary Table 1.

### Immunohistochemistry

Immunohistochemical staining was performed on 3-μm paraffinized sections of renal tissue. F4/80 (1:100, Abcam, Cambridge, UK), α-SMA (1:200, Abcam, Cambridge, UK), and PDGF-β (1:200, Abcam, Cambridge, UK) were detected in large-scale renal biopsies using an immunohistochemical kit (DAKO, Santa Clara, USA). Ten randomly selected nonoverlapping fields were captured for each slide and were analyzed using Image-Pro Plus software (Media Cybernetics, Rockville, MD, USA) in a blinded manner by two pathologists.

### ELISA assay

The concentration of KIM-1 (R&D，USA) in blood supernatants was quantified using an ELISA kit according to the manufacturer’s instructions.

### Western blotting

Renal tissues were homogenized in RIPA lysis buffer containing a protease inhibitor cocktail. Total protein concentrations were determined using a BCA assay kit following the manufacturer’s instructions. Sixty micrograms of protein were separated by sodium dodecyl sulphate–polyacrylamide gel electrophoresis, and the separated proteins were transferred to poly(vinylidene fluoride) membranes. The membranes were blocked with 5% nonfat milk in TBS with 0.1% Tween-20 for 1 h at 37°С and then probed with antibodies against TGF-β (1:500, Abcam, UK), IL-10 (1:200, Abcam, UK), TNF-α (1:200, Abcam, UK), and GAPDH (1:4000, Abbkine, China) at 4°С overnight. After being washed, the blots were incubated with an HRP-conjugated anti-IgG, and the target bands were visualized with ECL plus reagents following the manufacturer’s instructions. The signal intensity of the targeted band was quantified using ImageJ (NIH, USA).

### Statistical analyses

All results are presented as the mean  ±  SEM from at least three separate experiments. All data were statistically analyzed by GraphPad Prism software, version 5.0. We used a nonparametric Mann–Whitney U test or unpaired t test to evaluate *p* values. *p* < 0.05 was defined as statistically significant. n.s. *p* > 0.05, *p < 0.05, **p < 0.01, ***p < 0.001.

## Results

### Effect of clodronate liposomes on kidney weight and circulating monocytes and leukocytes

**Figure 1.**
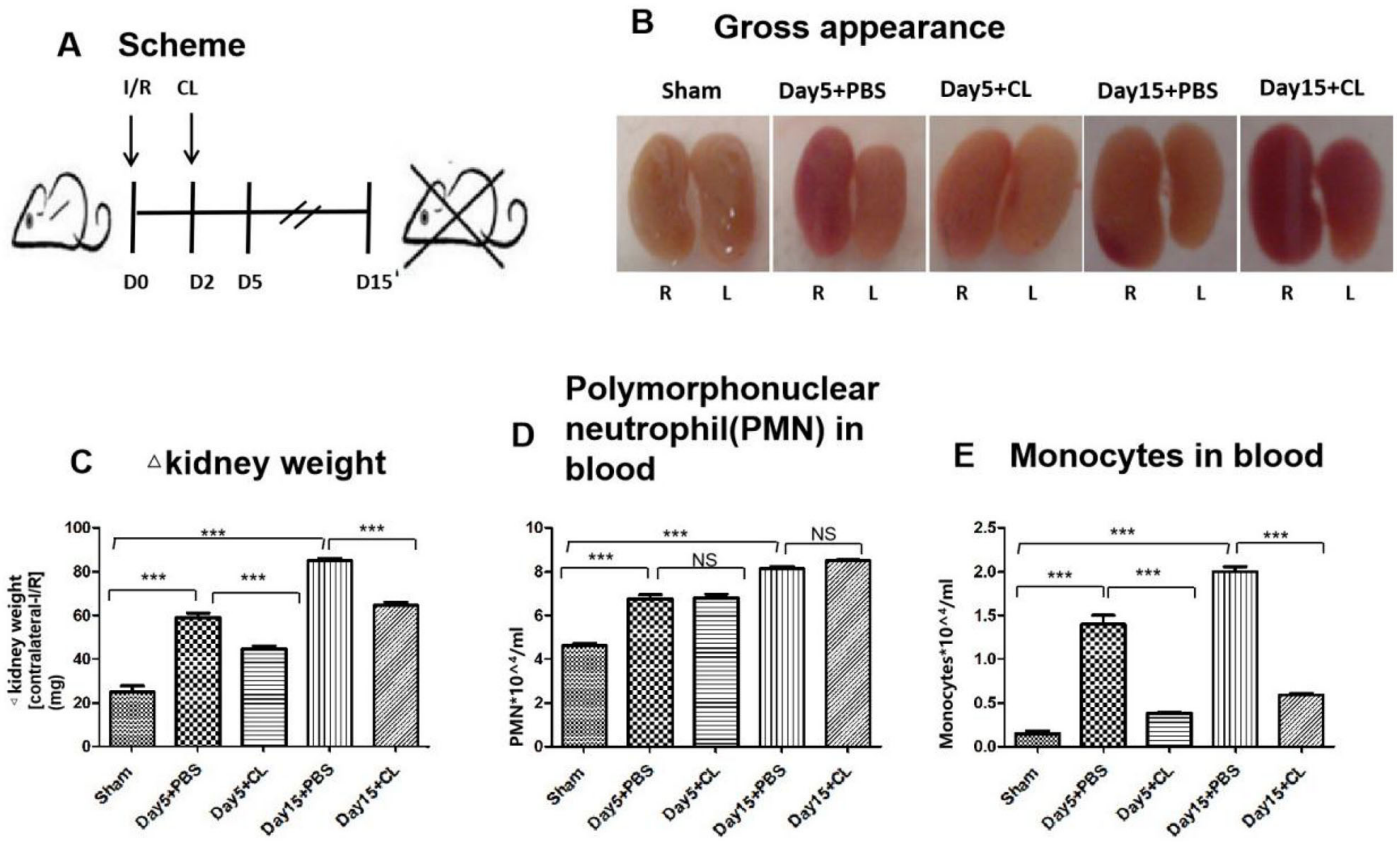
Effect of clodronate liposomes on kidney weight and circulating monocytes and leukocytes. (A) Scheme of the experimental plan. (B) Kidney gross appearance from mice in each group. (C) Statistical analysis of the change in kidney weights (contralateral minus I/R kidney). (D) Polymorphonuclear neutrophil cells (PMNs) in blood in different groups were analyzed by flow cytometry. (E) Monocytes in blood in different groups were analyzed by flow cytometry. n = 10 (***p < 0.001, **p < 0.005, *p < 0.05).

In this study, the I/R injury model was first established, and clodronate liposomes were injected into mice *via* the tail vein (Figure 1(A)). Kidney weight and size were more dramatically decreased in the clodronate liposomes treatment I/R group than in the PBS treatment group on Days 5 and 15 (Figure 1(B,C)). Polymorphonuclear neutrophil cells (PMNs) in blood in different groups were analyzed by flow cytometry, and there were no differences between the clodronate liposomes treatment group and the PBS treatment group (Figure 1(D)) on either Day 5 or Day 15 after I/R injury. However, monocytes in blood were decreased in the clodronate liposomes treatment group compared with the PBS treatment group (Figure 1(E)) on both Day 5 and Day 15 after I/R injury. Our data indicate that clodronate liposomes can reduce the number of monocytes in blood but have no effect on circulating leukocytes.

### Clodronate liposomes reduce the number of macrophages

**Figure 2.**
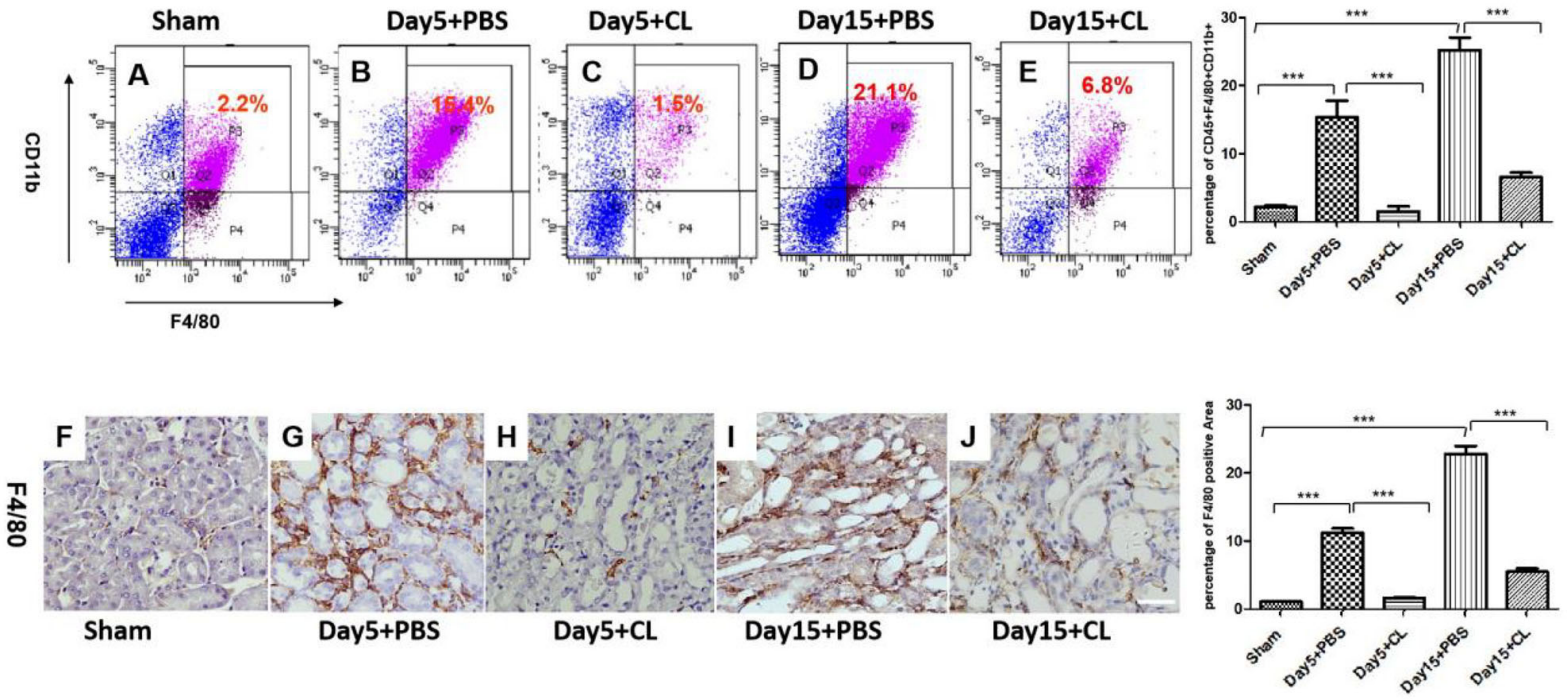
Clodronate liposomes reduced the number of macrophages in I/R injury. (A)–(E) CD45^+^F4/80^+^CD11b^+^cells in different groups by FACS; F4/80 (F)–(J) expression by IHC in different groups. *n* = 10 (***p < 0.001, **p < 0.005, *p < 0.05; scale bar= 100 μm).

The renal tissues of each group were digested for flow cytometry, and anti-mouse CD45, F4/80, and CD11b antibodies were used for labeling. The percentages of CD45^+^F4/80^+^CD11b^+^cells were 2.2% ± 0.4% (Figure 2(A)) in the sham group, 15.4% ± 4.2% (Figure 2(B)) in the Day 5 + PBS group, 1.5% ± 1.3% (Figure 2(C)) in the Day 5 + CL group, 21.1% ± 3.2% (Figure 2(D)) in the Day 15 + PBS group, 6.8% ± 2.1% (Figure 2(E)) in the Day 15 + CL group. The percentages of CD45^+^F4/80^+^CD11b^+^ cells were significantly decreased in the clodronate liposomes treatment group compared with the PBS treatment group after I/R injury (*p* < 0.005). Next, immunohistochemical detection of renal tissue showed that the positive rate of F4/80 expression was higher in the PBS treatment group than in the clodronate liposomes treatment group after I/R injury (Figure 2(F–J); *p* < 0.005).

### Clodronate liposomes attenuate I/R-induced tubulointerstitial damage and fibrosis

**Figure 3.**
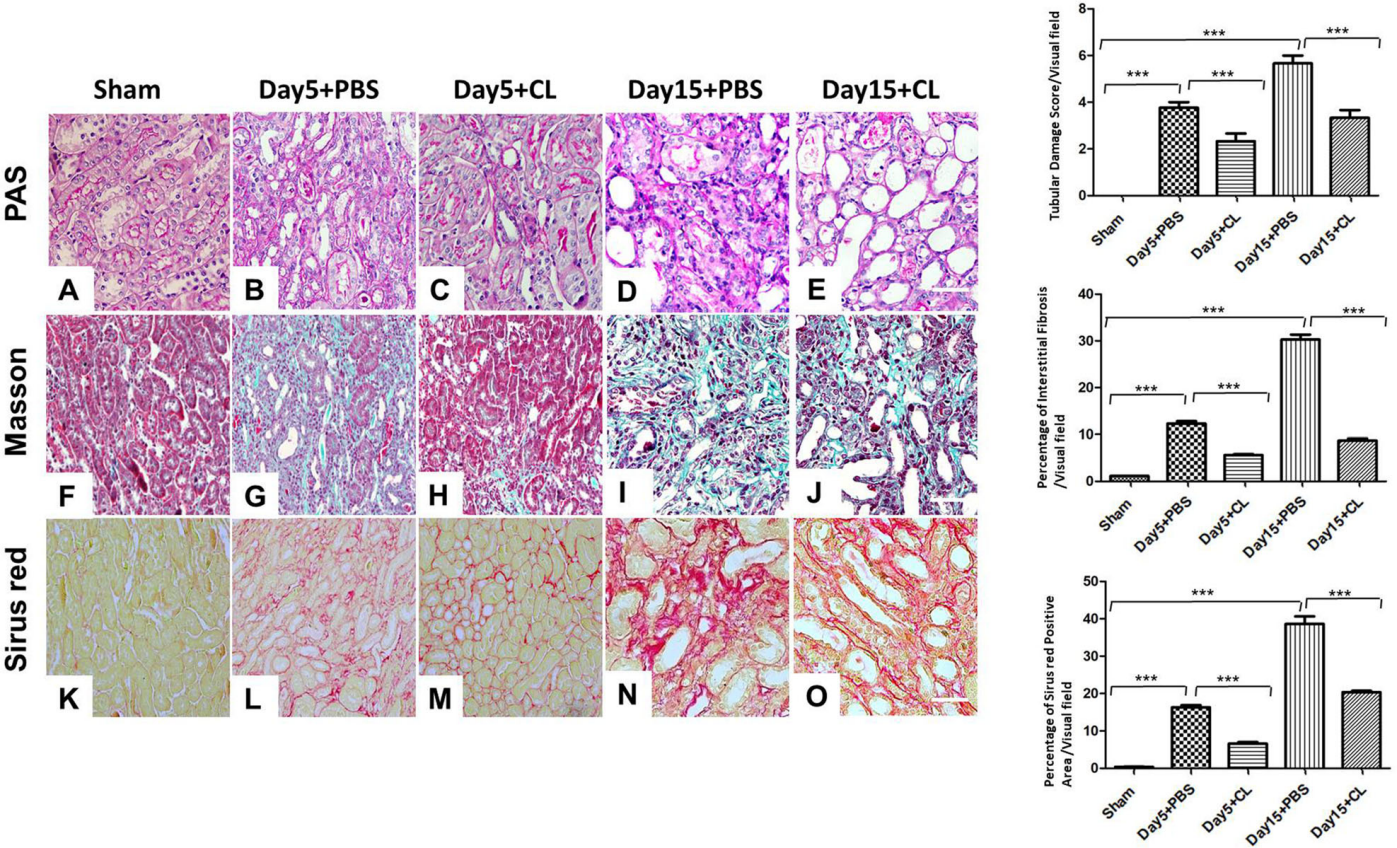
Clodronate liposomes attenuated I/R-induced tubulointerstitial damage and fibrosis. The renal tissue was embedded in conventional paraffin, sectioned at 3 μm, and stained with PAS (Figure 3(A–E)), Masson (Figure 3(F–J)), and Sirius red (Figure 3(K–O)). *n* = 10 (***p < 0.001, **p < 0.005, *p < 0.05; scale bar = 100 μm).

PAS (Figure 3(A–E)), Masson (Figure 3(F–J)), and Sirius red staining (Figure 3(K–O)) were used to evaluate the pathological injury and renal fibrosis. The renal tubular damage score in the Day 5 + PBS group was much higher than in the Day 5 + CL group (mean 3.8 vs. 2.1, *p* < 0.005). The renal tubular damage score in the Day 15 + PBS group was also much higher than in the Day 15 + CL group (mean 5.8 vs. 3.7, *p* < 0.005). In support of this finding, interstitial collagen deposition, as evaluated by Masson trichrome staining and Sirius red staining, was significantly decreased in the clodronate liposomes treatment group compared with the PBS treatment group on both Day 5 (mean 19.96 vs. 5.62 for Masson; 16.33 vs. 6.59 for Sirius red) and Day 15 (mean 30.3 vs. 8.71 for Masson; 38.6 vs. 20.3for Sirius red) after I/R injury. Taken together, our data indicate that clodronate liposomes treatment attenuates I/R-induced tubulointerstitial damage and fibrosis.

### Clodronate liposomes decrease α-SMA and PDGFR-β expression levels following I/R injury

**Figure 4.**
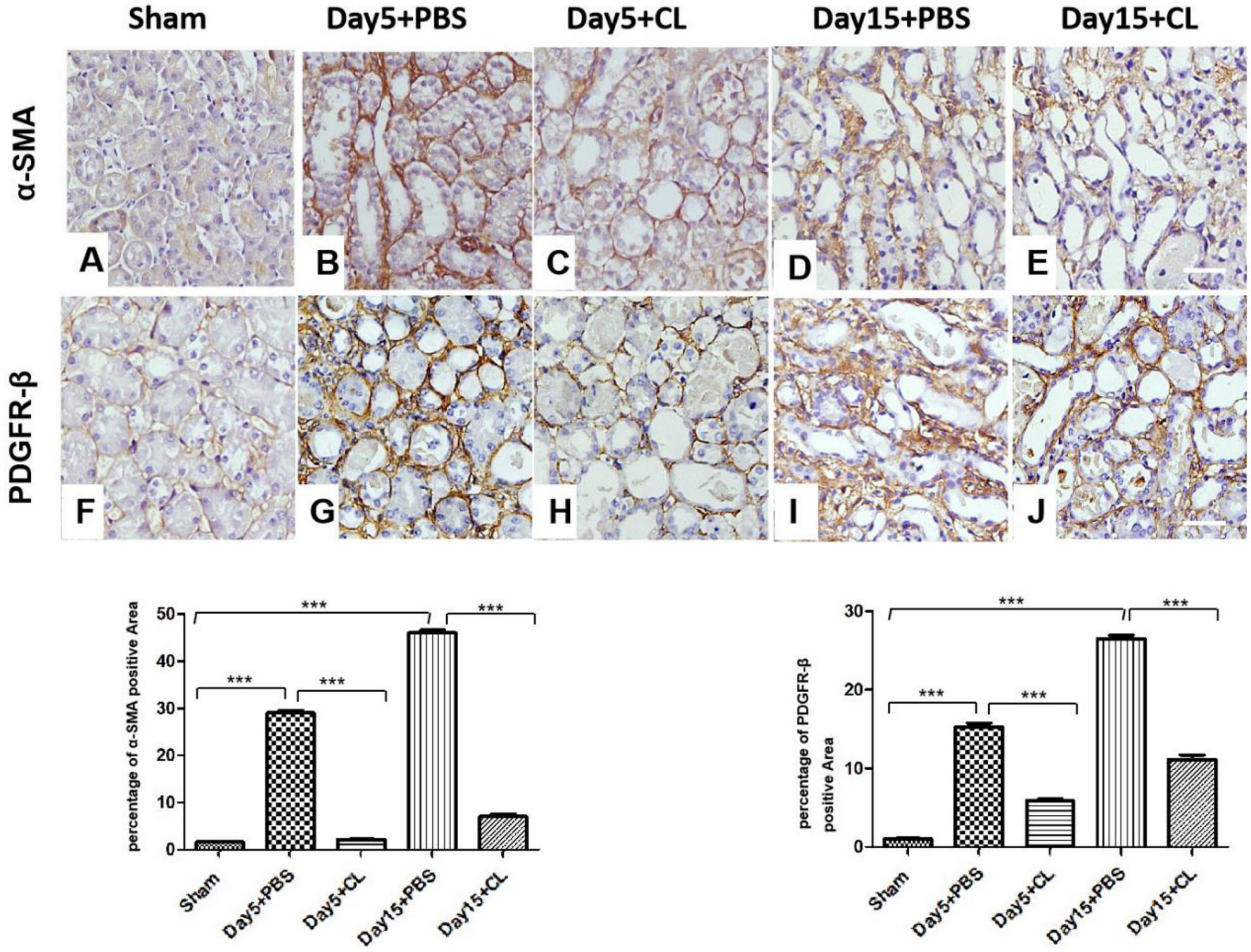
Clodronate liposomes decreased α-SMA and PDGFR-β expression levels following I/R injury. Immunohistochemical staining (IHC) was performed (magnification ×400). *n* = 10 (***p < 0.001, **p < 0.005, *p < 0.05; scale bar = 100 μm).

We also examined the effect of clodronate liposomes on the expression of the specific fibrosis markers α-SMA and PDGFR-β using immunohistochemistry (IHC). The immunohistochemistry results showed that the expressions of α-SMA (Figure 4(A–E)) and PDGFR-β (Figure 4(F–J)) in clodronate liposomes treatment group after I/R injury were significantly decreased compared with the PBS treatment group after I/R injury. As shown in Figure 4, the IHC analysis showed intense and more widely distributed expression of α-SMA (14.1-fold more than the Day 5 + CL group, 6.58-fold more than the Day 15 + CL group, *p* < 0.005), PDGFR-β (2.78-fold more than the Day 5 + CL group, 2.39-fold more than the Day 15 + CL group, *p* < 0.05) in the PBS treatment group on both Days 5 and 15. These data indicate that clodronate liposomes alleviate myofibroblasts and decrease renal interstitial fibrosis.

### Clodronate liposomes decrease α-SMA, fibronectin, and collagen I mRNA expression levels following I/R injury

**Figure 5.**
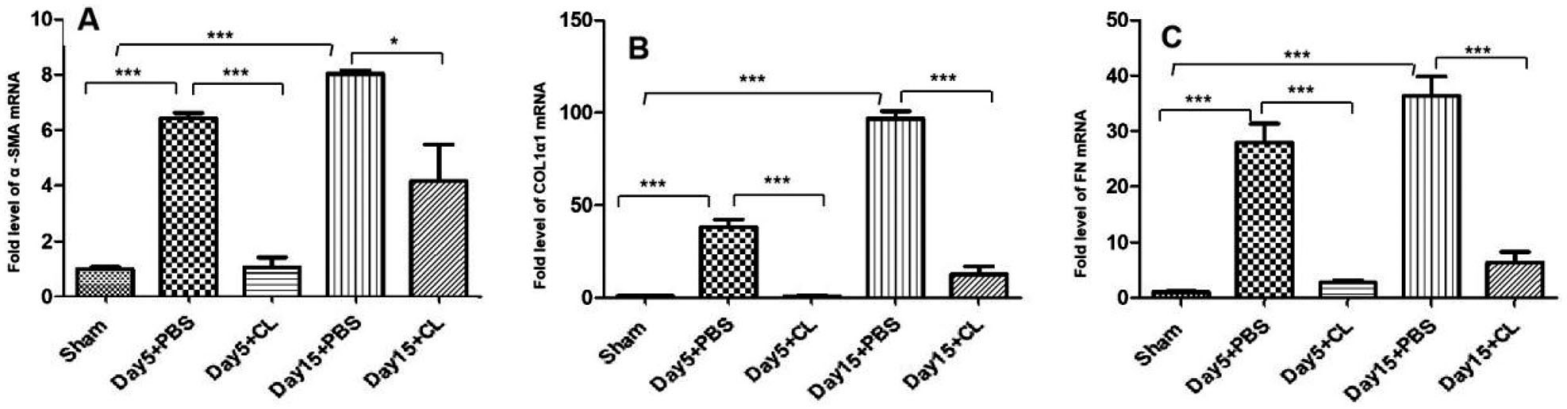
Clodronate liposomes decreased α-SMA, fibronectin and collagen I mRNA expression levels following I/R injury. α-SMA (A), collagen I (B), and fibronectin (C) mRNA expression were detected by RT–PCR in each group. *n* = 10 (***p < 0.001, **p < 0.005, *p < 0.05).

After injection of clodronate liposomes, the expression of α-SMA, fibronectin (FN) and collagen I was significantly decreased at the mRNA level. Compared with the Day 5 + PBS group, the expression of α-SMA mRNA was decreased five-fold in the Day 5 + CL group (Figure 5(A)). Compared with the Day 15 + PBS group, the expression of α-SMA mRNA was decreased two-fold in the Day 15 + CL group (Figure 5(A)). The expression of collagen I mRNA was decreased by 30-fold in the Day 5 + CL group compared with the Day 5 + PBS group (Figure 5(B)). The expression of collagen I mRNA was decreased by 50-fold in the Day 15 + CL group compared with the Day 15 + PBS group (Figure 5(B)). The expression of FN mRNA was decreased six-fold in the Day 5 + CL group compared with the Day 5 + PBS group (Figure 5(C)). The expression of FN mRNA was decreased fourfold in the Day 15 + CL group compared with the Day 15 + PBS group (Figure 5(C)).

### Clodronate liposomes decrease inflammatory cytokines secretion following I/R injury

**Figure 6.**
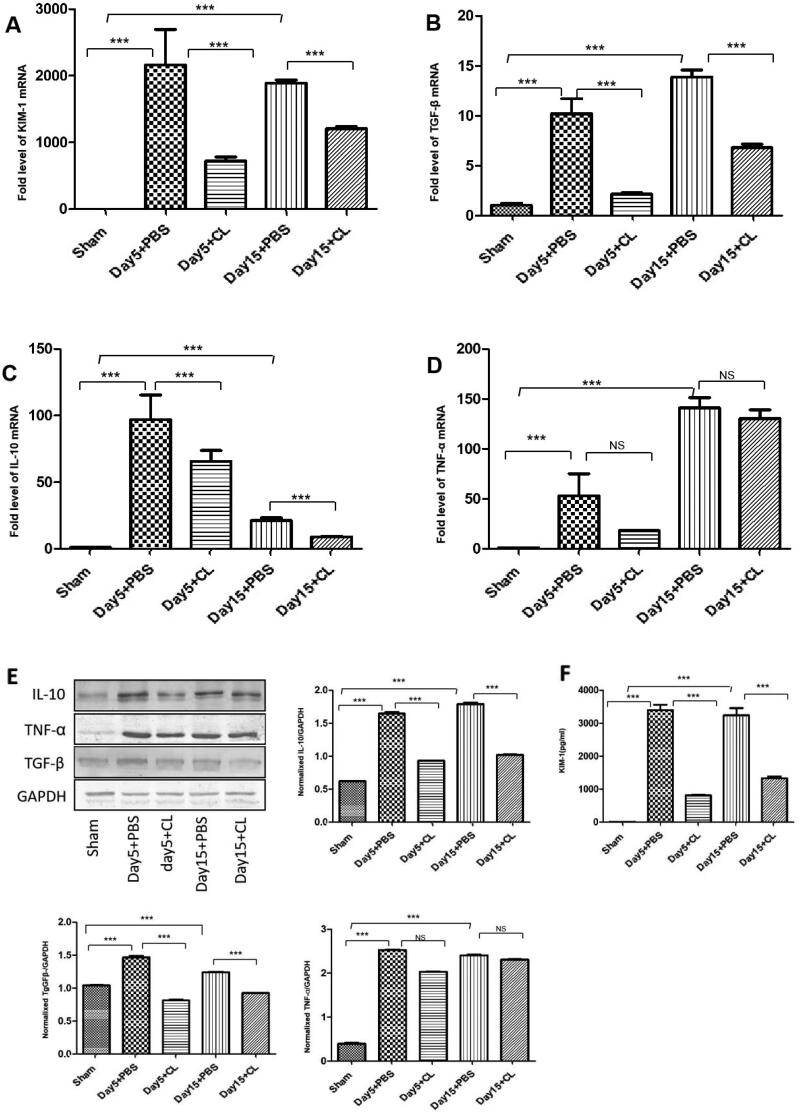
Clodronate liposomes decreased inflammatory cytokines secretion following I/R injury. KIM-1 (A), TGF-β (B), IL-10 (C) and TNF-α (D) mRNA expression were detected by RT–PCR in each group. IL-10 (E), TNF-α (E), and TGF-β (E)expression was detected by Western blotting in each group, KIM-1 (F) expression was detected by ELISA in each group. *n* = 10 (***p < 0.001, **p < 0.005, *p < 0.05).

After injection of the drug clodronate liposomes, the expression of KIM-1 (Figure 6(A,F)) was decreased in the clodronate liposomes treatment (Day 5 + CL and Day 15 + CL) groups compared with the PBS treatment (Day 5 + PBS and Day 15 + PBS) groups. The expression of TGF-β was decreased in the clodronate liposomes treatment (Day 5 + CL and Day 15 + CL) groups compared with the PBS treatment (Day 5 + PBS and Day 15 + PBS) groups (Figure 6(B,E)). The expression of IL-10 was decreased in the clodronate liposomes treatment (Day 5 + CL and Day 15 + CL) groups compared with the PBS treatment (Day 5 + PBS and Day 15 + PBS) groups (Figure 6(C,E)). While TNF-α was no difference was observed between in the clodronate liposomes treatment (Day 5 + CL and Day 15 + CL) groups and the PBS treatment (Day 5 + PBS and Day 15 + PBS) groups (Figure 6(D,E)) (**p < 0.01, ***p < 0.001, *p < 0.05).

## Discussion

In this study, we identified that depletion of macrophages by clodronate liposomes partially alleviated kidney fibrosis in renal I/R injury, possibly through the cytokines TGF-β and IL-10. Clodronate liposomes can reduce the number of monocytes in blood and macrophages in the kidney but have no effect on PMNs in blood. After the injection of clodronate liposomes, epithelial cell swelling, and cell shedding were reduced, and the collagen staining range was also reduced compared with the PBS treatment group. The expression levels of α-SMA, PDGF-β, fibronectin, collagen I, KIM-1, IL-10, and TGF-β were significantly decreased.

AKI is a very complex clinical disorder that is associated with severe morbidity and mortality. AKI is very common in hospitalized patients, especially critically ill patients. In our study, we established an I/R injury model in mice that progressed AKI to chronic kidney disease (CKD). After operation for 5 and 15 days, we found that the depletion of the macrophages by clodronate liposomes partially alleviated kidney fibrosis and injury.

Macrophages play a key role in the innate immune system with high plasticity and heterogeneity [14]. Macrophages are commonly classified into two distinct subsets. The first are classically activated or M1 macrophages, which are proinflammatory and polarized by lipopolysaccharide either alone or in association with Th1 cytokines such as IFN-γ and GM-CSF, and can produce proinflammatory cytokines such as interleukin-1β (IL-1β), IL-6, IL-12, IL-23, and TNF-α. Alternatively, activated or M2 macrophages, which are anti-inflammatory and immunoregulatory and polarized by Th2 cytokines such as IL-4 and IL-13, and can produce anti-inflammatory cytokines such as IL-10 and TGF-β [15]. When infection or inflammation is severe enough to affect an organ, macrophages can first exhibit the M1 phenotype to release TNF-α, IL-1β, IL-12, and IL-23 against the stimulus [16]. However, if the M1 phase continues, it can cause tissue damage [17]. Therefore, M2 macrophages can secrete high amounts of IL-10 and TGF-β to suppress inflammation, which contributes to remodeling, tissue repair, vasculogenesis, and the maintenance of homeostasis [15,18].

TGF-β is an important factor that promotes fibrosis or scar formation [19]. TGF-β is also associated with cellular proliferation, migration, differentiation, adhesion, tissue repair, angiogenesis immune surveillance, and survival [20]. Because M2 macrophages can produce TGF-β, it is thought that postischemic renal fibrosis serves as a major source of this profibrotic cytokine [21].

Clodronate liposome is a first-generation bisphosphonate drug encapsulated in a liposome used to deplete macrophages in animals. Previous studies have also shown that unselective macrophage depletion by clodronate liposomes administration can reduce experimental acute kidney damage [22]. Koichiro et al. [23] and Kim et al. [11] also showed that in a UUO model, clodronate liposomes can reduce renal fibrosis. In our study, we found that after injection of clodronate liposomes in the renal I/R injury model, the percentage of CD45^+^F4/80^+^CD11b^+^ cells was significantly decreased on Days 5 and 15. We also found that α-SMA, fibronectin, and collagen I expression was partly decreased (Figures 4 and 5), and KIM-1, IL-10, and TGF-β expression was also decreased (Figure 6). Additionally, the expression of KIM-1, a marker of renal injury [24], began to significantly increase on Day 5 in the PBS treatment group, and KIM-1 immuno-expression was seen mainly in the abnormally regenerating epithelial cells of damaged tubules. This finding indicates that after injection of clodronate liposomes, the macrophages in renal tissue were partly decreased, and renal fibrosis and renal injury were partially attenuated. Additionally, IL-10 and TGF-β expression in the clodronate liposomes treatment group was also decreased compared with that in the PBS treatment group. M2 macrophages secrete high amounts of IL-10 and TGF-β to suppress inflammation; therefore, the depletion of macrophages by clodronate liposomes may partially attenuate renal fibrosis because of M1/M2 polarization. In future studies, we will investigate which kinds of cells participate in the process.

In conclusion, we have demonstrated that the depletion of macrophages by clodronate liposomes can partially attenuate renal fibrosis in I/R injury through IL-10 and TGF-β, which may be correlated with M1/M2 polarization. In addition, macrophage depletion combined with cytotoxic therapies may be a promising new approach with high clinical potential in AKI and CKD.

## Supplementary Material

Supplemental Material
