# Supplementary material for: Depletion of macrophages with clodronate liposomes partially attenuates renal fibrosis on AKI–CKD transition
Source: Ren Fail. 2023 Jan 13;45(1):2149412. doi: 10.1080/0886022X.2022.2149412 (PMC9848250; doi:10.1080/0886022X.2022.2149412)
Supplement: Supplemental Material [file IRNF_A_2149412_SM7666.pdf]

Supplementary Material

1. Establishment of renal ischemia-reperfusion model

The results of PAS, Masson and Sirius red staining showed that in the IRI group, glomerular sclerosis was obvious, mesangial cell proliferation, tubular lumen expansion, tubular epithelial cell swelling or vacuolar degeneration, cell shedding, basement membrane exposure, and collagen staining range was significantly increased. Sirius red staining showed that the IRI+PBS group showed a significant increase in collagen (Fig1). The renal ischemia-reperfusion model was successfully established.

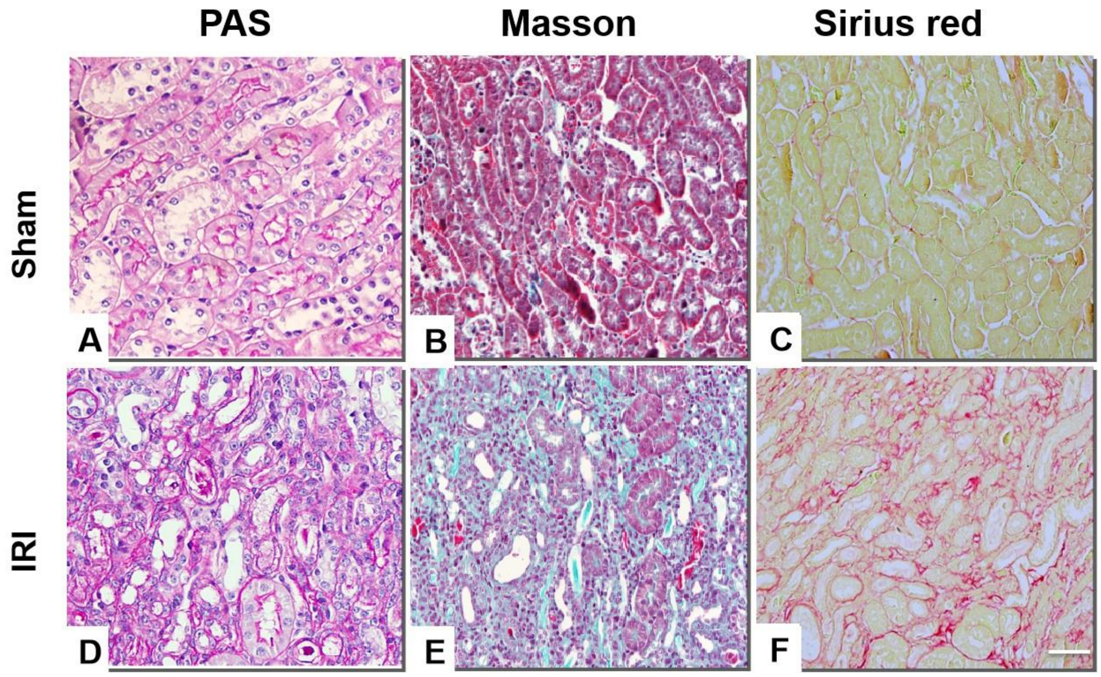

Figure 1. Histopathological changes in the kidneys of two groups of mice(×100)

Scale bar=100μm

2. Supplementary Table 1. Gene names and primer sequences used for qRT-PCR.

Gene Names	Primer Sequence
α-SMA	Germany,Qiagen，QT00140119

Information Classification: General

Fibronectin	Germany,Qiagen，QT00135758
CollagenI	Germany,Qiagen，QT00162204
TGF-β-F	CGCAACAACGCCATCTATGA
TGF-β-R	ACCAAGGTAACGCCAGGAAT
GAPDH	Germany,Qiagen,QT01658692
TNF-α	Germany,Qiagen,QT00104006
IL-10	Germany,Qiagen,QT00106169
Kim-1-F	TAAACCAGAGATTCCCACAC
Kim-1-R	GATCTTGTTGAAATAGTCGTGG

Information Classification: General
